# A phase II clinical trial of *Bifidobacterium longum subsp. longum* BL21 for preventing acute radiation enteritis among pelvic radiotherapy patients

**DOI:** 10.3389/fnut.2025.1626169

**Published:** 2025-11-10

**Authors:** Yinyin Yang, Xuhao Gu, Chang Liu, Li Zou, Liwei Xie, Yulong Liu, Peifeng Zhao, Ye Tian, Shang Cai

**Affiliations:** 1 Department of Radiotherapy and Oncology, The Second Affiliated Hospital of Soochow University, Suzhou, Jiangsu, China; 2 Department of Radiation Oncology, The Affiliated Wuxi People Hospital of Nanjing Medical University, Wuxi, Jiangsu, China; 3 Center of PRaG Therapy, Center for Cancer Diagnosis and Treatment, The Second Affiliated Hospital of Soochow University, Suzhou, Jiangsu, China

**Keywords:** pelvic radiotherapy, acute radiation enteritis, prevention, *Bifidobacterium longum subsp. longum* BL21, gut microbiota

## Abstract

**Background:**

Acute radiation enteritis (ARE) is a common side effect experienced by patients receiving pelvic radiotherapy (RT). Probiotic supplementation is an emerging strategy for preventing ARE.

**Methods:**

This phase II trial recruited patients with gynecologic or rectal cancers who received pelvic RT with curative or adjuvant intent. During RT, one packet of *Bifidobacterium longum subsp. longum* BL21 (BL21) powder was self-administered daily. ARE was assessed and classified according to the Radiation Therapy Oncology Group (RTOG) and Common Terminology Criteria for Adverse Events (CTCAE) toxicity grading criteria. We assessed the safety and efficacy of BL21 to prevent ARE and investigate the changes in the intestinal microbiota.

**Results:**

This study enrolled 52 patients, 8 participants withdrew, and 44 patients being included in the final analysis. The safety profile of BL21 during RT was favorable, and we did not observe any serious adverse events associated with BL21. Compared with our historical control data, these patients exhibited lower levels of ≥ grade 2 ARE and required fewer antidiarrheal medications (*n* = 12). Most diarrhea cases were classified as grade 1 (*n* = 22). Analysis of the gut microbiota revealed that the severity of ARE correlates with the abundance of BL21 and the increase in BL21 was associated with greater alpha diversity, an increase in beneficial bacteria, and a decrease in harmful bacteria.

**Conclusions:**

The favorable safety profile, exploratory clinical observations of reduced ARE severity vs. historical controls, and feasible administration support further investigation of *Bifidobacterium longum* BL21 as a prophylactic candidate for ARE, warranting validation in Phase III randomized controlled trials.

**Clinical Trial Registration:**

Chinese Clinical Study Registry (registry ID: ChiCTR2300069881).

## Background

Acute radiation enteritis (ARE) is a common side effect in patients undergoing radiotherapy (RT) for pelvic cancers that can result in malnutrition and potential interruption of RT, negatively affecting the prognosis of these patients. Several drugs, such as corticosteroids and non-steroidal anti-inflammatory agents, have been used to treat radiation-induced injuries. However, their protective effects against ARE remain unsatisfactory (1, 2).

Probiotics are defined as “live microorganisms that, when administered in adequate amounts, confer a health benefit to the host” (3). Supplementing with probiotics can be nutritionally beneficial to humans, particularly during the treatment of digestive system diseases (4). A recent study has underscored their significant relationship with the occurrence and progression of radiation-induced injuries in various organs, including the intestine (5). Throughout RT, intestinal microbiota diversity notably decreased, with a reduction in beneficial bacteria and an increase in harmful bacteria. Additionally, probiotic supplementation has demonstrated the ability to restore normal microbiota structure, providing a credible hypothesis that administering probiotics could attenuate ARE (6–8). Several preclinical studies, including ours, have demonstrated the potential efficacy of probiotics in preventing ARE. However, it remains unclear which specific probiotic species, strains, or combinations are optimal (9, 10).

*Bifidobacterium longum*, a gram-positive bacterium, was among the first microorganisms to colonize the human intestine. It belongs to the genus *Bifidobacterium* in the phylum Actinobacteria and forms a symbiotic relationship with the human body (11). Current research indicates that *Bifidobacterium longum* affects various aspects of human health throughout different life stages, impacting the digestive, immune, cardiovascular, and nervous systems (12). In digestive system disorders, *Bifidobacterium longum* shows promise in addressing intestinal inflammatory and is a potential radioprotective agent for preventing ARE (13).

*Bifidobacterium longum subsp. longum* BL21 (BL21), a subspecies of *Bifidobacterium longum*, exhibits potent free radical scavenging and antioxidant properties. It has demonstrated low toxicity and high safety in mouse models, with a median lethal dose exceeding 2 × 10^10^ CFU/kg (14). Our previous preclinical study demonstrated that BL21 prevents intestinal injury in a mouse model of total abdominal irradiation-induced ARE. Therefore, this Phase II pilot trial conducts initial evaluation of BL1′s safety profile and exploratory clinical outcomes related to ARE mitigation in pelvic irradiation patients, with findings requiring verification in subsequent controlled studies.

## Methods

### Participant eligibility

This single-arm, phase II study was registered in the Chinese Clinical Study Registry (registry ID: ChiCTR2300069881). Eligible patients (≥ 18 years) were newly diagnosed stage I-III gynecologic or rectal malignancies and were scheduled for pelvic radiotherapy with either curative or (neo) adjuvant intent. Patients were required to have a Karnofsky Performance Status (KPS) of ≥70 and adequate complete blood counts, hepatic reserve, and renal function for chemoradiotherapy. Those with a history of severe gastrointestinal disease or abnormal gastrointestinal function were excluded. Hospital Review Board approval was obtained before the screening and enrollment of all patients. All eligible patients signed an informed consent form after adequate communication before the study was initiated.

### Treatment schedule

The overall anticancer treatment schedule and indications for RT and chemotherapy for the enrolled patients adhered to the National Comprehensive Cancer Network guidelines. RT planning was conducted using computed tomography scans at 5 mm intervals across the entire volume of interest, with the patient positioned supine. The scans were performed following enema evacuation to improve the reliability of the rectal dose-volume histogram. The clinical target volume (CTV) was defined variably depending on the specific pathology; however, all definitions adhered to Radiation Therapy Oncology Group (RTOG) guidelines. The planning target volume (PTV) was defined as a uniform three-dimensional expansion of 5–7 mm around the CTV. Patients received 6MV intensity-modulated radiation therapy with fraction doses of 1.8–2 Gy administered once daily, 5 days a week. The prescription dose was 45–54 Gy at the PTV. The dose-volume constraints were followed for the organs at risk, mainly the large intestine, small intestine, rectum, and bladder, as routinely performed in clinical practice. All patients underwent bladder filling and rectal emptying before RT. The chemotherapy administered varied by cancer type: for cervical cancers, cisplatin was administered at a dose of 75–80 mg/m^2^ every 3 weeks, whereas for rectal cancers, either 5-fluorouracil was administered at 225 mg/m^2^ via continuous perfusion or capecitabine was provided in capsule form at a dose of 825–1,000 mg/m^2^ throughout the RT course.

### Study design

**Figure 1:**
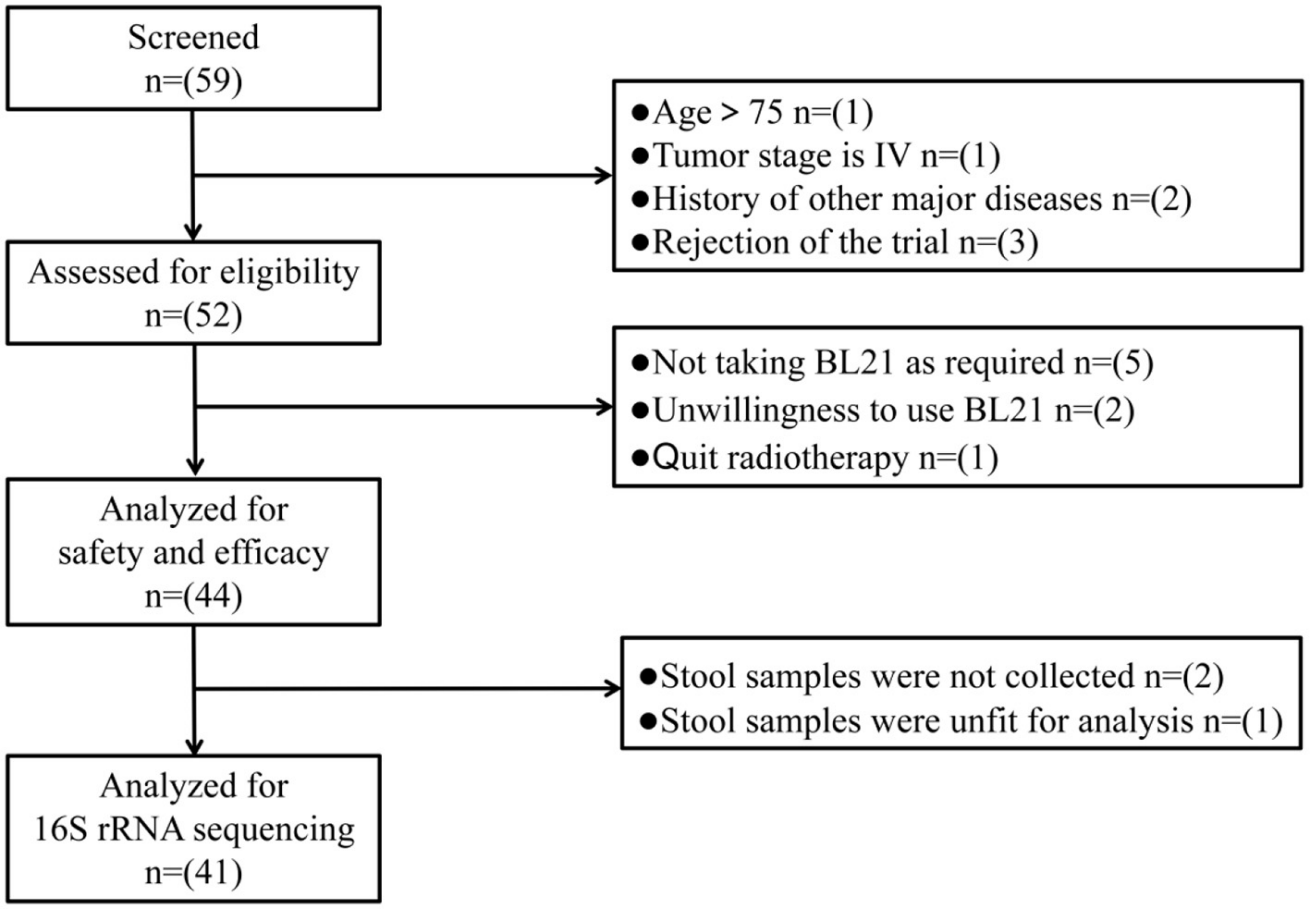
CONSORT (Consolidated Standards of Reporting Trials) diagram of the trial.

Eligible patients were instructed to self-administer one packet of BL21 powder orally daily from the 1st day of RT until completion. BL21 powder was produced and supplied by WeCare Probiotics Co., Ltd. (Suzhou, China) and manufactured in accordance with the Chinese Food Safety Enterprise Standard (ID: Q/WKSW0002S-2020). At the end of each week, patients were required to return all probiotic packaging bags, enabling the researchers to verify their adherence to the intervention protocol. Participants who did not take BL21 for more than 3 days were excluded from the study. During the 5–6 weeks of RT, dedicated follow-up personnel conducted weekly face-to-face interviews with patients. This personnel recorded various data points in a logbook, including patients' subjective symptoms, clinical signs, laboratory examinations, and medication usage. Stool samples were collected from patients on the first and final days of RT. These samples were immediately weighed and stored at −80 °C for future analysis using 16S rRNA gene sequencing. The flow chart of study procedure is presented in Figure 1.

### Outcome measurement

Researchers graded the severity of AEs and ARE during RT based on follow-up outcomes, with the highest grade representing the patient's final assessment. ARE was diagnosed and graded based on RTOG Lower gastrointestinal toxicity criteria. Grade 1, 2, 3, and 4 ARE were defined as increased frequency or change in quality of bowel habits not requiring medication/rectal discomfort not requiring analgesics, diarrhea requiring parasympatholytic drugs (e.g., Lomotil)/mucous discharge not necessitating sanitary pads/rectal or abdominal pain requiring analgesics, diarrhea requiring parenteral support/severe mucous or blood discharge necessitating sanitary pads/abdominal distention (flat plate radiograph demonstrates distended bowel loops), and acute or subacute obstruction, fistula or perforation; GI bleeding requiring transfusion; abdominal pain or tenesmus requiring tube decompression or bowel diversion, respectively. ARE and other AEs were also evaluated according to the Common Terminology Criteria for Adverse Events (CTCAE) Version 5.0 (15). The safety evaluation of BL21 focused on AEs, particularly abnormalities in liver and kidney functions, digestive discomfort, and weight changes. However, since RT can induce AEs, especially in the hematological and gastrointestinal systems, researchers investigated the relationship between the timing of these AEs and the administration of BL21. All AEs occurring during RT were reviewed by three senior radiotherapists or oncologists. Only AEs whose timing and frequency of occurrence were consistently deemed significantly related to the administration of BL21 were attributed to it. The severity of ARE was the primary endpoint, evaluated using the RTOG acute radiation morbidity scoring criteria (16). Additionally, diarrhea has been a key indicator for assessing the severity of intestinal radiation-induced injury in previous studies. Therefore, we evaluated the occurrence of diarrhea among patients to supplement our assessment of ARE.

### 16 S rRNA gene sequencing

Using fecal samples, 16S rRNA sequencing was performed using Metabo-Profile Biotechnology (Shanghai Co., Ltd., Shanghai, China), following the manufacturer's instructions. Initially, DNA was quantified using a Nanodrop, followed by a quality assessment of the DNA extraction using 1.2% agarose gel electrophoresis. Primers were designed to target microbial RNA or specific gene fragments, with sample-specific barcode sequences added for polymerase chain reaction (PCR) amplification of rRNA. The PCR-amplified products were quantified by fluorescence using the Quant-iT PicoGreen dsDNA Assay Kit as the fluorescent reagent and a microplate reader (BioTek, FLx800). Subsequently, sequencing libraries were prepared using the Illumina TruSeq Nano DNA LT Library Prep Kit, and high-throughput sequencing was performed using a sequencing machine. After obtaining the original data, preliminary screening was conducted to assess the sequence quality. Problematic samples were retested and supplemented. The QIIME2 dada2 analysis process or the analysis process of the Vsearch software was employed for sequence denoising or amplicon sequence variant (ASV) clustering. The specific composition of each sample at different taxonomic levels (kingdom, phylum, class, order, family, genus, and species) was determined to provide a holistic perspective.

### Statistical analysis

All statistical analyses were conducted using SPSS 25.0 software. Continuous variables are presented as medians (ranges). Categorical variables are reported as frequencies and percentages. The alpha diversity levels (Chao1 and Shannon) of each sample were evaluated based on the distribution of ASV in the different samples. Differences in the relative abundance of the gut microbiota between the two groups were analyzed using the rank sum test.

## Results

### Characteristics of participants

This study enrolled 52 patients between April and October 2023. Among the 8 patients who withdrew from the study, 5 failed to comply with the BL21 administration protocol, 2 withdrew due to personal reasons, and 1 was found to have a prior history of leukemia after enrollment. Finally, 44 patients (29 with gynecological cancer and 15 with rectal cancer) were included in the final analysis and their baseline characteristics were described in Table 1.

**Table 1:** Clinical, pathological, and treatment characteristics of patients.

**Characteristics**	***n* (%)**
Age, y	59.5 (Range, 35–74)
**Sex**	
Male	10 (22.7%)
Female	34 (77.3%)
**KPS status**	
70	15 (34.1%)
80	27 (61.4%)
90	2 (4.5%)
**Pathological**	
Cervical cancer	24 (54.5%)
Rectal cancer	15 (34.1%)
Endometrial cancer	5 (11.4%)
**Clinical stage**	
I	13 (29.5%)
II	15 (34.1%)
III	16 (36.4%)
**Concurrent chemotherapy**	
Yes	36 (81.8%)
No	8 (18.2%)
PTV dose, Gy	50 (Range, 41.6–54)

KPS, Karnofsky Performance Status; PTV, planning target volume.

### Safety assessment of BL21

All patients completed RT according to the original treatment plan; however, RT was interrupted in three patients. Of these, two patients experienced interruptions because of grade 3 or 4 febrile neutropenia, while another interrupted treatment for personal reasons. The primary AEs for all patients were hematological, with 22.7% experiencing grade 3 or 4 events, including anemia and decreased white blood cell and platelet count. However, no hepatic failure or renal injury was reported. Notably, all grade 3 or 4 hematological AEs occurred after chemotherapy and were attributed to chemoradiotherapy. Patients' body weights remained relatively stable, with only grade 1 and 2 weight loss observed, resulting in an incidence rate of 9.1% for both grades. Most individuals did not report any significant discomfort after BL21 administration. Only five patients reported experiencing grade 1 flatulence after taking BL21. This AE, likely related to BL21, was transient, resolved spontaneously without pharmacological intervention, and occurred only during the initial days of RT. Aside from diarrhea, no other grade 3 or 4 gastrointestinal AEs linked to BL21 have been reported. Therefore, BL21 is safe and reliable.

### Evaluation of ARE

Overall, based on RTOG riteria, the total incidence of ARE was 84.1%, and the incidences of grades 0–3 ARE were 15.9%, 56.8%, 22.7%, and 4.5%, respectively. None of the enrolled patients developed grade 4 ARE (Table 2, left). Notably, medical intervention was only required for ≥ grade 2 ARE according to the RTOG criteria. In this study, the ratio of ≥ grade 2 ARE was low at 27.2%, compared with that of our previous observational study, where the ratio was 65% (17). Meanwhile, based on CTCAE criteria, the incidence of grade 1, 2, and 3 diarrhea was 50%, 15.9%, and 13.6%, respectively, with no enrolled patients developing grade 4 diarrhea (Table 2, right). Additionally, the 12 patients who developed ≥ grade 2 ARE were the only ones who received antidiarrheal drugs, and five patients received antidiarrheal drugs only within a single week. The average time to antidiarrheal drug administration was approximately 25 days after RT initiation.

**Table 2:** Incidence and severity of acute radiation enteritis (left) and diarrhea (right).

**Grade (RTOG criteria)**	***N* (%)**	**Grade (CTCAE v5.0)**	***N* (%)**
0	7 (15.9%)	1	22 (50%)
1	25 (56.8%)	2	7 (15.9%)
2	10 (22.7%)	3	6 (13.6%)
3	2 (4.5%)	4	0 (0%)
4	0 (0%)	5	0 (0%)

RTOG, Radiation Therapy Oncology Group; CTCAE, Common Terminology Criteria for Adverse Events.

### Profiling of intestinal microbiota

**Figure 2:**
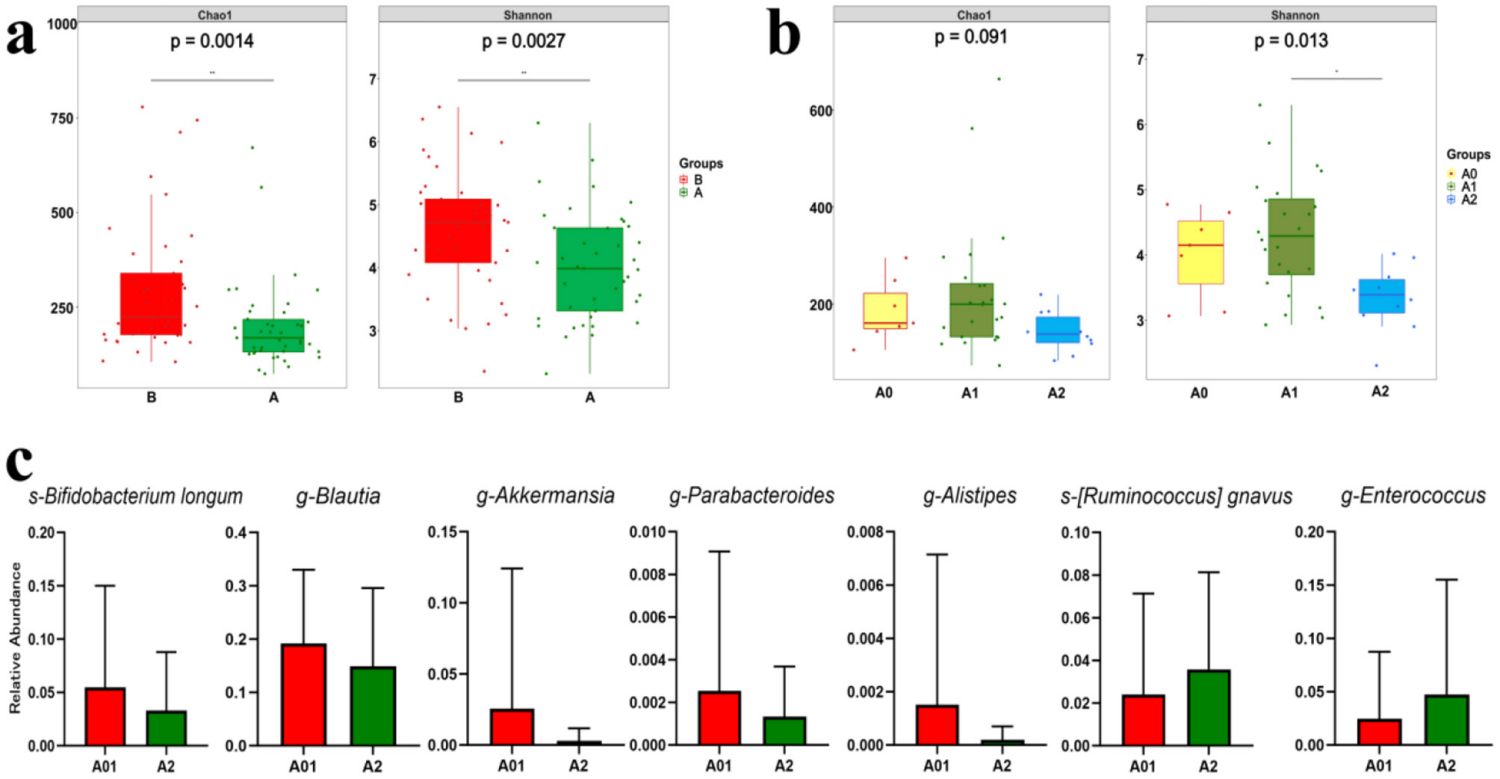
Alterations of gut microbiome. **(a)** Box plots of gut microbiota alpha diversity (Chao1 and Shannon indices) in patients pre- and post-radiotherapy. **(b)** Box plots comparing post-radiotherapy gut microbiota alpha diversity (Chao1 and Shannon indices) across ARE severity groups: severer (≥Grade 2, A2), mild (Grade 1, A1), and no symptoms (Grade 0, A0). **(c)** Box plots comparing post-radiotherapy relative abundance of specific beneficial and harmful bacteria across ARE severity groups: severer (≥Grade 2, A2), mild (Grade 1, A1), and no symptoms (Grade 0, A0). *p < 0.05; **p < 0.01; *p* < 0.05: statistically significant. ARE, acute radiation enteritis; RT, radiotherapy.

The fecal samples collected post-radiotherapy (group A) exhibited a sharp decline in gut microbiota alpha diversity compared to pre-RT samples (group B) (Figure 2a), demonstrating that pelvic RT significantly reduces microbial diversity and diminishes the heterogeneity of the gut microbiota community. According to the RTOG criteria, the symptoms of grade 1 ARE are mild and do not require specific treatment, patients were divided into severer ARE (≥ grade 2 ARE, group A2), mild ARE (grade 1 ARE, group A1), and none ARE (grade 0 ARE, group A0) groups for further analysis. As we can see, for fecal samples collected after RT, although no significant difference in gut microbiota alpha diversity was observed between the A0 and A1 groups (patients with no or mild ARE). However, the A2 group (severer ARE) showed significantly reduced alpha diversity (Shannon index) (Figure 2b). These findings suggest that abdominopelvic RT reduces both diversity and heterogeneity of the gut microbiota, while the BL21 probiotic may exert protective effects against ARE by enhancing microbial diversity and heterogeneity.

Although not representative of the actual relative abundance of the BL21 subspecies, our findings (Figure 2c) indicated that the relative abundance of *s-Bifidobacterium longum* was higher in the grades 0 and 1 ARE groups. This finding suggests that a greater presence of BL21 may be associated with patients exhibiting less severe ARE. The relative abundance of the genera *g-Blautia, g-Parabacteroides, g-Akkermansia*, and *g-Alistipes*, which are widely known for their benefits to host health, was higher in the grade 0 and 1 ARE group; however, it significantly decreased in ≥ grade 2 ARE group. Conversely, the relative abundance of pathogenic bacteria, such as *s-[Ruminococcus] gnavus* and *g-Enterococcus*, increased in the ≥ grade 2 ARE group but remained low in the grade 0 and 1 ARE group.

## Discussion

In this phase II single-arm clinical trial, we aimed to preliminarily evaluate the safety and potential efficacy of BL21 in preventing ARE. First, BL21 demonstrates a consistent safety profile, both in gynecological and rectal cancer patients, that parallels findings in mouse models (14). We instructed patients to take one packet of probiotic powder containing 20 billion CFU of BL21 daily orally and we did not observe any serious AEs associated with BL21. According to the RTOG criteria, grade 1 ARE presents with mild symptoms, while medical intervention is required only for grade 2 or higher ARE. Our results demonstrated that after administration of the probiotic BL21, the incidences of grade 0/1 and grade ≥2 ARE were 72.7% and 27.2%, respectively. As this was a single-arm phase II clinical trial, we compared these outcomes with historical control data from our institution (17). The observational clinical study referenced indicated that among abdominopelvic cancer patients receiving radiotherapy in our department, the incidences of grade 0/1 and grade ≥2 ARE were 35% and 65%, respectively. Consequently, this current study provides exploratory clinical observations that prophylactic use of BL21 may reduc ARE severity in pelvic radiotherapy patients compared to our historical controls.

The ability of many probiotic species to protect the intestinal mucosa from lesions caused by irradiation in mice has been investigated (10, 18). However, these outcomes yield different results in clinical practice. A randomized, double-blind, placebo-controlled study utilized a combination of *Lactobacillus acidophilus* LA-5 and *Bifidobacterium animalis subsp*. lactis BB-12 to prevent ARE. The probiotic group had a significantly lower incidence of grades 1 and 2 diarrhea (52.8%) than the placebo group (82.1%). Additionally, 50.0% of patients in the probiotic group required antidiarrheal drugs, compared to 85.7% in the placebo group (19). Demers et al. used two different probiotic dose regimens of *Lactobacillus acidophilus* LAC-361 and *Bifidobacterium longum* BB-536 mixed: a standard dose of 1.3 billion CFU twice a day and a high dose of 10 billion CFU daily. However, the overall incidence of diarrhea was high (86%), and no significant differences were observed between the groups using the World Health Organization grading for diarrhea and the National Cancer Institute grading for abdominal pain. The proportion of patients who received antidiarrheal drugs was 42.5%, 30.2%, and 27.4% in the placebo, standard-dose probiotic, and high-dose probiotic groups, respectively (20). In another study, probiotic capsules containing live *Lactobacillus acidophilus* and *Bifidobacterium bifidum* were used. Despite both groups experiencing a 100% rate of National Cancer Institute grades of diarrhea, participants in the probiotics group had a notable decrease in the incidence of grades 2 and 3 diarrhea. Additionally, 32% of the patients in the placebo group required antidiarrheal drugs compared to only 9% of those in the probiotic group (21). The timing of the onset of antidiarrheal drug intervention reflects the severity of ARE. Delia et al. reported the shortest time interval. The average time of loperamide use from the start of RT in the probiotic and placebo groups was 5.08 days and 3.58 days, respectively (22). In other studies, the duration for both groups was approximately 20 days (19, 20).

Our results showed that the overall incidence of diarrhea was 76.9%, this figure was lower than the 86% reported by Demers et al. (20) and 100% reported by Chitapanarux et al. (21). The incidence of grades 1 and 2 diarrhea in this study was 65.9%, similar to that reported by Linn et al. (19). Among the 29 patients who experienced grade 1 and 2 diarrhea, most (22 patients) had only grade 1 diarrhea, indicating mild symptoms. Clinicians evaluated the need for antidiarrheal drugs by considering ARE severity in patients receiving pelvic RT. Typically, individuals who required medical intervention were categorized as having ≥ grade 2 ARE following the RTOG criteria (15). In our study, 12 patients (27.2%) experienced ≥ grade 2 ARE; only these patients used antidiarrheal drugs during RT, which was lower compared to the placebo group (29.27%−85.7%) in the previous studies and consistently lower than those reported in probiotic groups (9%−50%) except for the study by Chitapanarux et al. (19–21, 23). The time between antidiarrheal drug use and the beginning of RT was 25 days, longer than the approximately 20 days reported in other studies (19, 20).

Additionally, we focused on the alterations in the intestinal microbiota of patients using 16S rRNA sequencing. To our knowledge, studies have investigated either the effects of probiotics on preventing ARE or the impact of RT on the intestinal microbiota, with few reporting specific changes in the intestinal microbiota resulting from probiotics use to prevent ARE. RT has been shown to affect the diversity and composition of the intestinal microbiota in patients with pelvic cancer. A 2008 study involving 10 patients with pelvic cancer suggested that the intestinal microbiota could serve as a potential predictive marker for the occurrence and severity of ARE (24). With the emergence of 16S rRNA sequencing technology, a study reported that patients who experienced ARE during RT exhibited lower alpha diversity, relatively higher abundances of Proteobacteria and Gammaproteobacteria, and lower abundances of Bacteroides (25). Another study found that the diversity of the intestinal microbiota decreased during RT and established a linear relationship between higher intestinal microbiota diversity and improved gastrointestinal function (6). Additionally, our previous study demonstrated that an increased abundance of pathogenic bacteria and a decreased abundance of beneficial bacteria are other characteristics of ARE (7).

In this study, we observed a decrease in alpha diversity post-RT, and patients with ≥ grade 2 ARE exhibited lower alpha diversity compared to those with grade 0 and 1 ARE. This illustrates the accuracy of our ARE classification, as previous studies have demonstrated a linear relationship between the severity of ARE and alpha diversity (6). We also observed differences in intestinal microbiota composition between patients with ≥ grade 2 ARE and those with grades 0 and 1. The relative abundance of s*-Bifidobacterium longum* was higher in grade 0 and 1 ARE groups, suggesting that the relative abundance of BL21 is associated with ARE severity. Specifically, patients exhibiting higher levels of BL21 in the gut tended to have lower ARE severity. In addition, we noted that the relative abundances of several beneficial bacteria were higher in the grade 0 and 1 ARE groups, while the relative abundances of several harmful bacteria were lower compared to the ≥ grade 2 ARE group. We hypothesize that this change may be attributed to *Bifidobacterium longum*, which has been demonstrated to reduce harmful bacteria while promoting the growth of beneficial bacteria (26, 27). For example, g-Blautia, crucial in maintaining intestinal microbiota, enhancing intestinal barrier function, and mitigating intestinal inflammation and infection, was higher in the grade 0 and 1 ARE groups (28, 29). Meanwhile, Fang et al. demonstrated that *Bifidobacterium longum* CCFM1029 exerted beneficial effects on atopic dermatitis, potentially by maintaining Lachnospiraceae abundance and reshaping the intestinal microbiota. This finding aligns with the results of this study, as g-Blautia is a genus within the Lachnospiraceae family (30). Conversely, s-[Ruminococcus] gnavus, associated with inflammatory bowel disease and gut inflammation, was higher in the ≥ grade 2 ARE group (31). Therefore, we hypothesize that the prevention of ARE by *Bifidobacterium longum* may be associated with the regulation of intestinal microbiota, but further validation in subsequent studies is warranted.

Our findings should be interpreted cautiously because of the single-arm study design and the limited number of patients. None of the observed differences in the intestinal microbiota composition reached statistical significance; therefore, our results should indicate a trend. This limitation may be attributed to the small sample size and the considerable influence of environmental and dietary factors on intestinal bacteria. Therefore, designing randomized controlled trials is crucial to validate these findings. Further investigation is warranted to understand how BL21 mitigates ARE while controlling for various confounding factors, including pathology, chemotherapy, and dietary habits. However, analyzing actual clinical data in conjunction with comparisons to other studies and the role of the intestinal microbiota may provide valuable insights to address the objectives of this research.

## Conclusions

Our study demonstrates that oral BL21 administration in pelvic radiotherapy patients was safe and clinically feasible, with no serious adverse events observed. Preliminary efficacy data suggest potential protective effects against ARE when compared to historical controls, as evidenced by: (1) reduced incidence of ≥grade 2 ARE, (2) decreased antidiarrheal medication requirements, and (3) predominance of grade 1 diarrhea cases. Microbiome analysis further indicates that BL1′s possible ARE prevention mechanism may involve gut microbiota modulation. However, these preliminary findings warrant validation through randomized controlled trials to establish BL1′s efficacy and mechanistic basis.

## Data Availability

The data presented in this study are publicly available. The data can be found here: https://db.cngb.org/download, accession CNP0008304.

## References

[1] Klopp AH Yeung AR Deshmukh S Gil KM Wenzel L Westin SN . Patient-reported toxicity during pelvic intensity-modulated radiation therapy: NRG Oncology-RTOG 1203. J Clin Oncol. (2018) 36:2538–44. doi: 10.1200/JCO.2017.77.4273 29989857 PMC6097832

[2] Wang K Tepper JE. Radiation therapy-associated toxicity: etiology, management, and prevention. CA Cancer J Clin. (2021) 71:437–54. doi: 10.3322/caac.21689 34255347

[3] Hill C Guarner F Reid G Gibson GR Merenstein DJ Pot B . Expert consensus document. The International Scientific Association for Probiotics and Prebiotics consensus statement on the scope and appropriate use of the term probiotic. Nat Rev Gastroenterol Hepatol. (2014) 11:506–14. doi: 10.1038/nrgastro.2014.66 24912386

[4] Faccio AA Mattos CHPS Santos EASD Neto NRM Moreira RP Tonelo Batella L . Oral nutritional supplementation in cancer patients who were receiving chemo/chemoradiation therapy: a multicenter, randomized phase II study. Nutr Cancer. (2021) 73:442–9. doi: 10.1080/01635581.2020.1758170 32363940

[5] Wang W Cui B Nie Y Sun L Zhang F. Radiation injury and gut microbiota-based treatment. Protein Cell. (2024) 15:83–97. doi: 10.1093/procel/pwad044 37470727 PMC10833463

[6] Mitra A Grossman Biegert GW Delgado AY Karpinets TV Solley TN Mezzari MP . Microbial diversity and composition is associated with patient-reported toxicity during chemoradiation therapy for cervical cancer. Int J Radiat Oncol Biol Phys. (2020) 107:163–71. doi: 10.1016/j.ijrobp.2019.12.040 31987960 PMC7932475

[7] Zhao TS Xie LW Cai S Xu JY Zhou H Tang LF . Dysbiosis of gut microbiota is associated with the progression of radiation-induced intestinal injury and is alleviated by oral compound probiotics in mouse model. Front Cell Infect Microbiol. (2021) 11:717636. doi: 10.3389/fcimb.2021.717636 34760714 PMC8573182

[8] Acharya M Venkidesh BS Mumbrekar KD. Bacterial supplementation in mitigation of radiation-induced gastrointestinal damage. Life Sci. (2024) 353:122921. doi: 10.1016/j.lfs.2024.122921 39032692

[9] Xie LW Lu HY Tang LF Tang FL Zhu RQ Wang DF . Probiotic consortia protect the intestine against radiation injury by improving intestinal epithelial homeostasis. Int J Radiat Oncol Biol Phys. (2024) 120:189–204. doi: 10.1016/j.ijrobp.2024.03.003 38485099

[10] Riehl TE Alvarado D Ee X Zuckerman A Foster L Kapoor V . Lactobacillus rhamnosus GG protects the intestinal epithelium from radiation injury through release of lipoteichoic acid, macrophage activation and the migration of mesenchymal stem cells. Gut. (2019) 68:1003–13. doi: 10.1136/gutjnl-2018-316226 29934438 PMC7202371

[11] Bottacini F Van Sinderen D Ventura M. Omics of bifidobacteria: research and insights into their health-promoting activities. Biochem J. (2017) 474:4137–52. doi: 10.1042/BCJ20160756 29212851

[12] Mills S Yang B Smith GJ Stanton C Ross RP. Efficacy of *Bifidobacterium longum* alone or in multi-strain probiotic formulations during early life and beyond. Gut Microbes. (2023) 15:2186098. doi: 10.1080/19490976.2023.2186098 36896934 PMC10012958

[13] Li M Ding J Stanton C Ross RP Zhao J Yang B . *Bifidobacterium longum subsp.* infantis FJSYZ1M3 ameliorates DSS-induced colitis by maintaining the intestinal barrier, regulating inflammatory cytokines, and modifying gut microbiota. Food Funct. (2023) 14:354–68. doi: 10.1039/D2FO03263E 36511157

[14] Dong Y Zhang Y Xu F Zou K. Extensive genomic characterization, pre-clinical probiotic evaluation, and safety analysis of *Bifidobacterium longum subsp. longum* BL21 isolated from infant feces. Microb Pathog. (2024) 197:107100. doi: 10.1016/j.micpath.2024.107100 39505088

[15] Common Terminology Criteria for Adverse Events (CTCAE) v5.0. National Institutes and health, National cancer Institute 2017, 2017. National Institutes and Health. Available online at: https://ctep.cancer.gov/protocolDevelopment/electronic_applications/ctc

[16] Cox JD Stetz J Pajak TF. Toxicity criteria of the Radiation Therapy Oncology Group (RTOG) and the European Organization for Research and Treatment of Cancer (EORTC). Int J Radiat Oncol Biol Phys. (1995) 31:1341–6. doi: 10.1016/0360-3016(95)00060-C 7713792

[17] Cai S Yang Y Kong Y Guo Q Xu Y Xing P . Gut bacteria *Erysipelatoclostridium* and its related metabolite Ptilosteroid A could predict radiation-induced intestinal injury. Front Public Health. (2022) 10:862598. doi: 10.3389/fpubh.2022.862598 35419331 PMC8995795

[18] Lee YS Kim TY Kim Y Lee SH Kim S Kang SW . Microbiota-derived lactate accelerates intestinal stem-cell-mediated epithelial development. Cell Host Microb. (2018) 24:833–46.e6. doi: 10.1016/j.chom.2018.11.002 30543778

[19] Linn YH Thu KK Win NHH. Effect of probiotics for the prevention of acute radiation-induced diarrhoea among cervical cancer patients: a randomized double-blind placebo-controlled study. Probiotics Antimicrob Proteins. (2019) 11:638–47. doi: 10.1007/s12602-018-9408-9 29550911

[20] Demers M Dagnault A Desjardins J. A randomized double-blind controlled trial: impact of probiotics on diarrhea in patients treated with pelvic radiation. Clin Nutr. (2014) 33:761–7. doi: 10.1016/j.clnu.2013.10.015 24200199

[21] Chitapanarux I Chitapanarux T Traisathit P Kudumpee S Tharavichitkul E Lorvidhaya V. Randomized controlled trial of live Lactobacillus acidophilus plus *Bifidobacterium bifidum* in prophylaxis of diarrhea during radiotherapy in cervical cancer patients. Radiat Oncol. (2010) 5:31. doi: 10.1186/1748-717X-5-31 20444243 PMC2874795

[22] Delia P Sansotta G Donato V Frosina P Salatino A Messina G . Use of probiotics for prevention of radiation-induced diarrhea. World J Gastroenterol. (2007) 13:912–5. doi: 10.3748/wjg.v13.i6.912 17352022 PMC4065928

[23] Giralt J Regadera JP Verges R Romero J de la Fuente I Biete A . Effects of probiotic *Lactobacillus casei* DN-114 001 in prevention of radiation-induced diarrhea: results from a multicenter, randomized, placebo-controlled nutritional trial. I*nt J Radiat Oncol Biol Phys*. (2008) 71:1213–9. doi: 10.1016/j.ijrobp.2007.11.009 18243569

[24] Manichanh C Varela E Martinez C Antolin M Llopis M Doré J . The gut microbiota predispose to the pathophysiology of acute postradiotherapy diarrhea. Am J Gastroenterol. (2008) 103:1754–61. doi: 10.1111/j.1572-0241.2008.01868.x 18564125

[25] Wang Z Wang Q Wang X Zhu L Chen J Zhang B . Gut microbial dysbiosis is associated with development and progression of radiation enteritis during pelvic radiotherapy. J Cell Mol Med. (2019) 23:3747–56. doi: 10.1111/jcmm.14289 30908851 PMC6484301

[26] Toscano M De Grandi R Stronati L De Vecchi E Drago L. Effect of *Lactobacillus rhamnosus* HN001 and *Bifidobacterium longum* BB536 on the healthy gut microbiota composition at the phyla and species level: a preliminary study. World J Gastroenterol. (2017) 23:2696–704. doi: 10.3748/wjg.v23.i15.2696 28487606 PMC5403748

[27] Jo SH Jeon HJ Song WS Lee JS Kwon JE Park JH . Unveiling the inhibition mechanism of Clostridioides difficile by *Bifidobacterium longum* via multiomics approach. Front Microbiol. (2023) 14:1293149. doi: 10.3389/fmicb.2023.1293149 38029200 PMC10663266

[28] Ozato N Saito S Yamaguchi T Katashima M Tokuda I Sawada K . *Blautia* genus associated with visceral fat accumulation in adults 20-76 years of age. NPJ Biofilms Microbiomes. (2019) 5:28. doi: 10.1038/s41522-019-0101-x 31602309 PMC6778088

[29] Hosomi K Saito M Park J Murakami H Shibata N Ando M . Oral administration of *Blautia* wexlerae ameliorates obesity and type 2 diabetes via metabolic remodeling of the gut microbiota. Nat Commun. (2022) 13:4477. doi: 10.1038/s41467-022-32015-7 35982037 PMC9388534

[30] Fang Z Pan T Li L Wang H Zhu J Zhang H . *Bifidobacterium longum* mediated tryptophan metabolism to improve atopic dermatitis via the gut-skin axis. Gut Microbes. (2022) 14:2044723. doi: 10.1080/19490976.2022.2044723 35239463 PMC8903757

[31] Schirmer M Garner A Vlamakis H Xavier RJ. Microbial genes and pathways in inflammatory bowel disease. Nat Rev Microbiol. (2019) 17:497–511. doi: 10.1038/s41579-019-0213-6 31249397 PMC6759048

